# Retraction Note: Mast cells are the main interleukin 17-positive cells in anticitrullinated protein antibody-positive and -negative rheumatoid arthritis and osteoarthritis synovium

**DOI:** 10.1186/s13075-015-0847-3

**Published:** 2015-12-09

**Authors:** Jolien Suurmond, Annemarie L. Dorjée, Mariëtte R. Boon, Edward F. Knol, Tom W. J. Huizinga, René E. M. Toes, Annemie J. M. Schuerwegh

**Affiliations:** Department of Rheumatology, Leiden University Medical Center, PO Box 9600, Albinusdreef 2, C1-R, NL-2300 RC Leiden, The Netherlands; Department of Dermatology/Allergology, University Medical Center Utrecht, Heidelberglaan 100, NL-3584 CX Utrecht, The Netherlands

## Retraction

The authors retract this article [1] following an investigation by Leiden University Medical Centre into the research activities of the last author. The investigation identified a discrepancy between the data reported in the article and the original collected data. The investigation committee concluded that this undermined the scientific basis of the publication and advised that the publication should be retracted.

The online version of this article contains the full text of the retracted article as electronic supplementary material (Additional file 1).

## Additional file

Additional file 1:
**Mast cells are the main interleukin 17-positive cells in anticitrullinated protein antibody-positive and -negative rheumatoid arthritis and osteoarthritis synovium. **(PDF 1320 kb)
